# How salary satisfaction and working conditions shape professional attitudes of primary school teachers in Vietnam: a structural equation modeling analysis during the 2024–2026 salary reform

**DOI:** 10.3389/fpsyg.2026.1862300

**Published:** 2026-10-07

**Authors:** Thi Quynh Mai Le, Yi Jiang Li

**Affiliations:** 1 School of Educational Science, Anhui Normal University, Wuhu, Anhui, China; 2 Huaibei Normal University, Huaibei, Anhui, China

**Keywords:** primary school teachers, salary satisfaction, SEM, teaching attitudes, Vietnam education policy, working conditions

## Abstract

**Introduction:**

Vietnam faces a severe teacher shortage (over 102,000 positions), partly due to low salaries and high work pressure. The 2024–2026 salary reform is intended to address these issues, but its association with teachers' professional attitudes remains underexplored.

**Methods:**

This study examines how salary satisfaction and working conditions relate to the professional attitudes of primary school teachers in Vietnam during the salary reform period. A cross-sectional survey of 357 public primary school teachers was conducted. Data were analyzed using Structural Equation Modeling (SEM) to test the associations between salary satisfaction, working conditions, actual income, fairness in salary policy, and professional attitudes.

**Results:**

Salary satisfaction had the strongest positive association with professional attitudes (β = 0.631, *p* < 0.001). Working conditions also showed a positive association (β = 0.346, *p* =0.022); because working conditions were reverse-coded so that higher scores indicate more positive conditions, this finding indicates that teachers who report more positive working conditions tend to show more positive professional attitudes. Actual income (β = 0.038, *p* =0.818) and fairness in salary policy (β = −0.139, *p* =0.301) were not significantly associated with salary satisfaction, and neither was significantly associated with professional attitudes (all *p* >0.05).

**Discussion:**

In conclusion, salary satisfaction and working conditions were the two key correlates of professional attitudes in this sample. Policy implications may include increasing basic salaries, reconsidering the proposed abolition of seniority allowances, and improving working conditions in disadvantaged areas. Given the cross-sectional design, all findings are interpreted as associations rather than causal effects.

## 1 Introduction

### 1.1 Research background

Globally, teacher shortages have become a critical issue in many developing countries (World Bank, 2018; Glewwe and Muralidharan, 2016). According to the Ministry of Education and Training of Vietnam (MOET, 2025), the country is facing a shortage of over 102,000 teachers, including more than 30,000 preschool teachers and 72,000 general education teachers, with approximately 60,000 approved teaching positions remaining unfilled. This shortage is particularly severe in primary education, where the workload is high and salaries remain low. According to OECD (2025), teacher salaries and working conditions remain key policy levers for attracting and retaining qualified teachers. The Law on Education (Qu&##7889;c h&##7897;i, 2019) and its 2025 amendment Qu&##7889;c h&##7897;i (2025) provide the legal framework for teacher salary reform.

In response, the Vietnamese government has implemented a comprehensive salary reform based on Resolution No. 27-NQ/TW (2018) and the Law on Teachers (2025). Under this reform, teacher salaries are positioned at the highest level in the public administrative salary scale. According to the Ministry of Education and Training's Action Plan No. 2811/QÐ-BGDÐT (October 10, 2025), from January 1, 2026, teacher salaries will be calculated using the formula:

**Salary** **=** **Basic salary** **×Current salary coefficient** **×** **Special coefficient**

The special coefficient for primary and secondary school teachers who hold a bachelor's degree is approximately 1.15. Additionally, teachers in particularly disadvantaged areas, border regions, islands, and ethnic minority areas will receive allowances of up to 100%.

However, despite these policy efforts, actual implementation has faced significant challenges. Teachers with under 10 years' experience report that their basic salaries remain insufficient to cover living costs, especially in large cities where rent consumes 40–50% of their income (I-GV1, male, 28 years old, Hanoi). The situation has been further complicated by Circular No. 29/2024/TT-BGDÐT (B&##7897;, 2024), which prohibits primary-level tutoring, cutting off a major source of supplementary income for many teachers. According to survey data, after accounting for tutoring income, the proportion of teachers with total monthly income exceeding 20 million VND increased from 9.5 to 23.5%, indicating heavy reliance on non-salary income sources.

### 1.2 Theoretical foundation

This study draws on two complementary theoretical frameworks.

First, public policy theory provides a lens for understanding the gap between policy design and implementation. According to Chen (2003); Chen and Garbe (2011), policy analysis must consider not only the content of policies but also the context, actors, and implementation mechanisms. Chen et al. (2024) further emphasize that “policy implementation gaps” often arise due to unclear guidance, delayed funding, or administrative inconsistencies. These theoretical insights are particularly relevant for analyzing Vietnam's salary reform, where teachers express cautious optimism (SAT4R = 2.31, indicating low trust) and a “wait-and-see” attitude.

Second, motivation theory helps explain how salary and working conditions relate to teachers' attitudes and behaviors. Herzberg's two-factor theory (Herzberg, 1959) distinguishes between hygiene factors (such as salary and working conditions) and motivators (such as recognition and achievement). According to this theory, inadequate salary can cause dissatisfaction, but improving salary alone may not necessarily lead to motivation. Vroom's expectancy theory (Vroom, 1964) further suggests that teachers' motivation depends on their expectation that effort will lead to performance and that performance will lead to rewards. Adams' equity theory (Adams, 1965) adds that teachers compare their input-output ratio with that of others, and perceived inequity can lead to dissatisfaction.

These theoretical frameworks have been applied in previous studies of teacher motivation in Vietnam. Bui and Dinh (2025) examined factors influencing high school students' choice of pedagogical majors, finding that intrinsic motivation (love for children, passion for teaching) was the strongest predictor. Lê et al. 2025c) studied teacher salary satisfaction in Vietnam, finding that satisfaction levels remained low despite policy improvements, primarily due to the gap between basic salary and living costs.

### 1.3 Research gap and objectives

While previous studies have examined teacher salary and job satisfaction in various contexts (Hanushek, 2011; Skaalvik and Skaalvik, 2011; Toropova et al., 2021), limited research has been conducted on the relationship between salary satisfaction, working conditions, and professional attitudes of primary school teachers in Vietnam during a major salary reform period. Specifically, no study has used Structural Equation Modeling (SEM) to analyze these relationships in the context of the 2024–2026 salary reform and the ban on primary tutoring (Circular No. 29/2024/TT-BGDÐT).

This study has three main objectives: First, it examines how salary satisfaction relates to the professional attitudes of primary school teachers. Second, it examines the role working conditions play in shaping those attitudes. Finally, it explores how pressure from parents and a sense of fairness when comparing themselves to others relate to both salary satisfaction and teachers' professional attitudes.

**Research questions:**

1. Is salary satisfaction significantly associated with teachers' professional attitudes?

2. Are working conditions and parental pressure significantly associated with teachers' professional attitudes?

3. Do comparative fairness play significant roles in these relationships?

## 2 Literature review

### 2.1 Public policy theory and salary reform

Public policy theory provides a framework for understanding the design, implementation, and evaluation of salary policies. According to Chen (2003), policy analysis involves examining the context, actors, and processes that shape policy outcomes. Chen and Garbe (2011) emphasizes that effective policy implementation requires clear goals, adequate resources, and coordination among stakeholders. Chen et al. (2024) specifically focus on policy implementation gaps—the discrepancies between policy intentions and actual outcomes. They identify several factors that contribute to these gaps: unclear policy guidelines, delayed or insufficient funding, administrative inconsistencies across regions, lack of monitoring mechanisms, and resistance from implementing agents. These theoretical insights are highly relevant to Vietnam's salary reform. Teachers are aware of the policy but have not yet experienced its tangible benefits, which reflects a classic “policy implementation gap.”

### 2.2 Teacher salary and job satisfaction

International research has consistently shown that salary is a significant predictor of teacher job satisfaction and retention. Hanushek (2011) argues that higher teacher salaries attract more qualified individuals into the profession and reduce turnover. Ingersoll and Smith (2003) found that low salary is one of the main reasons teachers leave the profession, especially in the first five years of teaching. Skaalvik and Skaalvik (2011) examined teacher job satisfaction and motivation to leave the teaching profession in Norway, finding that salary satisfaction was a significant predictor of both job satisfaction and turnover intentions. Toropova et al. (2021) analyzed TALIS data and found that working conditions—including workload, administrative support, and professional autonomy—were even more important than salary in predicting teacher job satisfaction. In the Vietnamese context, Lê (2025a,b); Lê et al. (2025c) studied teacher salary satisfaction and found that satisfaction levels remained low despite policy improvements. Ma and Lei (2025) examined compensating teachers serving rural schools in southern China and found that targeted financial incentives helped attract and retain teachers in disadvantaged areas. Their survey of 450 teachers across three provinces revealed that the majority of teachers (67.5%) reported being “dissatisfied” or “very dissatisfied” with their current salary, primarily due to the gap between basic salary and living costs. The study also Arnold and Rahimi (2025) found that teachers in urban areas were less satisfied than those in rural areas, due to higher housing and transportation costs. Martins and Ferreira (2025) analyzed individual incentive reforms in the public sector and showed that teacher pay reforms can have significant effects on performance and retention. Johnston (2025) examined preferences, selection, and the structure of teacher pay, showing that pay design influences who enters and stays in teaching. Thompson-Lee et al. (2025) systematically reviewed STEM teacher recruitment and retention interventions, highlighting the role of financial incentives and working conditions. Liu and Yang (2026) found that salaries significantly affect the well-being of university teachers in China. Lê (2025a) and Lê (2025b) studied job satisfaction and career events among Vietnamese teachers, finding that salary and working conditions were central concerns.

### 2.3 Working conditions and teacher stress

Teacher stress and burnout have been extensively studied in international literature. Kyriacou (2001) defines teacher stress as “the experience by a teacher of unpleasant, negative emotions, such as anger, anxiety, tension, frustration or depression, resulting from some aspect of their work.” Maslach and Leiter (2016) identify three dimensions of burnout: emotional exhaustion, depersonalization, and reduced personal accomplishment. Hakanen et al. (2006) found that job demands (such as workload and time pressure) were associated with burnout, while job resources (such as social support and autonomy) were associated with work engagement. Collie et al. (2012) found that school climate and social-emotional learning significantly influenced teacher stress and job satisfaction. In Vietnam, the Journal of Education (2023) reported that primary school teachers face multiple sources of stress: (1) high workload (teaching multiple subjects, preparing lessons, grading papers, handling administrative tasks), (2) pressure from parents (increasing expectations, complaints, and even lawsuits), (3) pressure from educational reforms (the 2018 General Education Program), and (4) pressure from performance-based competition (“achievement pressure”). The report noted that many teachers work 10–12 h per day, including evenings and weekends. Arnold and Rahimi (2025) found that teachers' working conditions and wellbeing were key factors associated with intention to leave the profession.

### 2.4 Professional attitudes and career commitment

Professional attitudes refer to teachers' beliefs, feelings, and behavioral intentions toward their profession. These include pride in being a teacher, intention to stay in the profession, willingness to recommend the profession to others, and motivation to improve teaching practices. Klassen and Chiu (2010) found that teachers' self-efficacy and job satisfaction were positively associated with their commitment to the teaching profession. Liu and Ramsey (2008) analyzed teacher follow-up survey data and found that salary, working conditions, and administrative support were significant predictors of teacher retention. In Vietnam, Bui and Dinh (2025) found that intrinsic motivation (love for children, passion for teaching) was the strongest predictor of high school students' choice of pedagogical majors. However, the Journal of Education (2023) reported that among in-service teachers, the willingness to recommend the teaching profession to others was low (only 2.66 out of 5), suggesting a gap between pre-service motivation and in-service attitudes.

### 2.5 Conceptual framework and hypotheses

Based on the theoretical framework and literature review, this study proposes a conceptual model that includes five key constructs. Each construct is clearly defined below. The conceptual framework and hypothesized relationships are illustrated in Figure 1.

**Figure 1:**
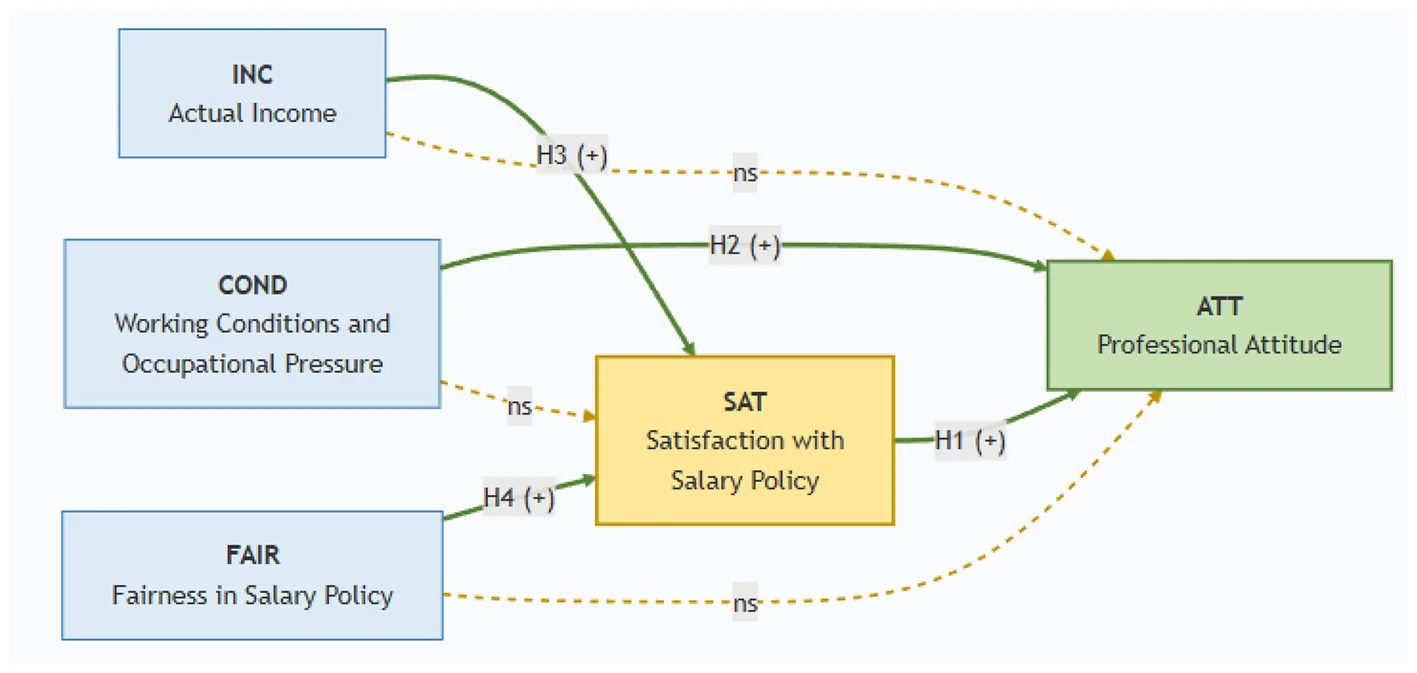
Conceptual framework and hypothesized relationships.

#### 2.5.1 Professional attitudes (ATT)

Teachers' beliefs, feelings, and behavioral intentions toward their profession, including pride in being a teacher, intention to remain in teaching, willingness to recommend the profession, and motivation to teach (5 items: ATT1–ATT5).

#### 2.5.2 Satisfaction with salary policy (SAT)

Teachers' satisfaction with salary policies, including perceptions of reasonableness, adequacy, pension security, trust in future reforms, and feeling valued by the state. (5 items: SAT1–SAT5).

#### 2.5.3 Working conditions and occupational pressure (COND)

Teachers' perceptions of their workload, unpaid overtime, administrative tasks, parental pressure, and curriculum reform pressure. These items were reverse-coded so that higher scores indicate more negative working conditions. (5 items: COND1R–COND5R).

#### 2.5.4 Actual income (INC)

Teachers' perceptions of income adequacy, savings ability, and the need for supplementary income (5 items: INC1–INC5).

#### 2.5.5 Fairness in salary policy (FAIR)

Teachers' perceptions of how fair their pay is when compared with people in other professions and whether salary reflects their workload and contributions. (5 items: FAIR1–FAIR5).

From this model, we propose the following hypotheses:

- **H1:** Satisfaction with salary policy (SAT) has a positive association with professional attitudes (ATT).
- **H2:** Working conditions and occupational pressure (COND) have a positive association with professional attitudes (ATT).
- **H3:** Actual income (INC) has a positive association with satisfaction with salary policy (SAT).
- **H4:** Fairness in salary policy (FAIR) has a positive association with satisfaction with salary policy (SAT).

## 3 Methodology

### 3.1 Sample and data collection

A cross-sectional survey was conducted with 357 public primary school teachers from four regions of Vietnam (Region 1: 30.8%, Region 2: 24.9%, Region 3: 27.7%, Region 4: 16.5%). The sample included teachers from urban, rural, and mountainous areas, ensuring regional representation. The demographic characteristics of the sample are presented in Table 1.

**Table 1:** Demographic characteristics of the sample (*N* = 357).

Characteristic	Category	*n*	%
Gender	Female	258	72.3
	Male	99	27.7
Age	Under 30	96	26.9
	30–39	111	31.1
	40–49	82	23.0
	50+	68	19.0
Education	College	40	11.2
	Bachelor's	270	75.6
	Postgraduate	47	13.2
Region	Region 1	110	30.8
	Region 2	89	24.9
	Region 3	99	27.7
	Region 4	59	16.5
School location	Urban	142	39.8
	Rural	128	35.9
	Mountainous	87	24.4
Tenure	1–5 years	78	21.8
	6–10 years	95	26.6
	11–20 years	112	31.4
	20+ years	72	20.2

Source: Survey data, 2026.

#### 3.1.1 Recruitment procedure

Participants were recruited through a combination of purposive and snowball sampling. Initial contact was made with school principals in each region, who distributed the survey link to eligible teachers. Eligibility criteria included: (1) currently employed as a full-time primary school teacher in public schools, (2) at least 1 year of teaching experience, and (3) willingness to participate voluntarily.

#### 3.1.2 Missing data treatment

Missing data were minimal (< 2% per item). Little's MCAR test indicated that data were missing completely at random (χ^2^ = 45.32, df = 38, *p* = 0.192). Missing values were handled using maximum likelihood estimation in AMOS 24.0.

#### 3.1.3 Data availability

The datasets generated and analyzed during the current study are available from the corresponding author on reasonable request.

### 3.2 Instruments

Five scales were developed based on the literature review and adapted to the Vietnamese context. All items were measured on a 5-point Likert scale (1 = strongly disagree to 5 = strongly agree). Reverse-coded items were used where appropriate.

#### 3.2.1 Actual income (INC) scale

5 items (INC1–INC5) measuring teachers' perceptions of income adequacy, savings ability, and the need for supplementary income.

#### 3.2.2 Fairness in salary policy (FAIR) scale

5 items (FAIR1–FAIR5) measuring teachers' perceptions of fairness in salary policies, including comparison with other professions and seniority-based increments.

#### 3.2.3 Working conditions and occupational pressure (COND) scale

5 reverse-coded items (COND1R–COND5R) measuring workload, administrative tasks, parental pressure, and curriculum reform pressure. Higher scores indicate more negative working conditions.

#### 3.2.4 Satisfaction with Salary Policy (SAT) Scale

5 items (SAT1–SAT5) measuring satisfaction with salary policies, including pension security and trust in future reforms.

#### 3.2.5 Professional Attitude (ATT) Scale

5 items (ATT1–ATT5) measuring intention to stay in the profession, pride in being a teacher, willingness to recommend the profession to others, and teaching motivation under current salary levels.

### 3.3 Validity and reliability

Cronbach's Alpha was calculated for each scale to assess internal consistency.

All scales met the acceptable threshold of α > 0.6, with SAT and ATT showing excellent reliability (α > 0.9). However, the FAIR scale showed relatively low reliability (α = 0.630), which should be noted as a limitation. Cronbach's alpha for each scale is reported in Table 2.

**Table 2:** Reliability statistics for the scales.

Scale	Cronbach's α	Items
INC (Actual Income)	0.689	5
FAIR (Fairness in Salary Policy)	0.630	5
COND (Working Conditions and Occupational Pressure)	0.734	5
SAT (Satisfaction with Salary Policy)	0.921	5
ATT (Professional Attitude)	0.949	5

Source: Reliability analysis, 2026.

#### 3.3.1 Exploratory factor analysis (EFA)

EFA was conducted to examine the factor structure. The KMO measure was 0.854, and Bartlett's test of sphericity was significant (χ^2^ = 5938.314, df = 300, *p* < 0.001), indicating suitability for factor analysis. Five factors were extracted with eigenvalues >1, explaining 62.148% of the total variance. Factor loadings ranged from 0.460 to 0.925.

#### 3.3.2 Justification for using the same sample for EFA and CFA

EFA revealed a factor structure that differed from the original questionnaire design. Specifically, COND and FAIR items loaded onto overlapping factors, and some items (e.g., INC2, INC4R, FAIR1, FAIR2, FAIR3, FAIR5) showed cross-loadings. Given the sample size of 357, splitting the sample would have resulted in insufficient statistical power for both EFA and CFA. Hair et al. (2010) suggest that a minimum of 5–10 observations per estimated parameter is required. With 25 observed variables and 5 latent constructs, the sample size meets this requirement. However, we acknowledge that using the same sample for EFA and CFA may capitalize on chance. As a robustness check, we conducted a split-sample cross-validation (odd-even split), and the pattern of factor loadings and model fit was consistent across both subsamples.

#### 3.3.3 Confirmatory factor analysis (CFA)

CFA was conducted to validate the measurement model.

#### 3.3.4 Model fit indices

CMIN/df = 3.782, CFI = 0.873, TLI = 0.856, RMSEA = 0.088 (90% CI: 0.082–0.094), SRMR = 0.078. While CFI and TLI were below the ideal threshold of 0.90, the model was considered acceptable given the complexity of the constructs, the sample size, and the fact that RMSEA and SRMR were within acceptable ranges (Hair et al., 2010). Modification indices were examined, and no correlated errors were added because the theoretical model did not justify them.

#### 3.3.5 Discriminant validity

Discriminant validity was assessed by comparing the square root of AVE for each construct with the correlations between constructs. For SAT and ATT, the square root of AVE exceeded the inter-construct correlations, indicating good discriminant validity. However, for COND, INC, and FAIR, the square root of AVE was lower than some inter-construct correlations, suggesting potential discriminant validity issues. This is acknowledged as a limitation.

### 3.4 Data analysis

Data were analyzed using SPSS 26.0 and AMOS 24.0. The analysis proceeded in four steps: (1) Descriptive statistics and reliability analysis. (2) Exploratory Factor Analysis (EFA) to examine factor structure. (3) Confirmatory Factor Analysis (CFA) to validate the measurement model. (4) Structural Equation Modeling (SEM) to test the hypothesized relationships. Given the cross-sectional design, all relationships are interpreted as associations rather than causal effects.

## 4 Results

### 4.1 Descriptive statistics

The descriptive statistics reveal a contrast: teachers report relatively positive professional attitudes (ATT variables), with ATT1 (stay until retirement) at 3.91 and ATT4 (proud to be a teacher) at 3.84. However, their satisfaction with salary and working conditions is low. Most INC items are below 3.00, with INC2 (salary covers family expenses) at 2.63 and INC4R (need extra work to survive) at 2.34. Fairness perceptions (FAIR) are also low, except for FAIR4 (seniority-based pay) at 3.35. All COND variables are below 2.00, with COND2R (unpaid overtime) at 1.54 and COND4R (too many non-teaching tasks) at 1.76. Descriptive statistics for all observed variables are presented in Table 3.

**Table 3:** Descriptive statistics for observed variables.

Variable	Item description	Mean	SD
INC1	Salary is sufficient for personal expenses	2.92	1.283
INC2	Salary is sufficient for family expenses	2.63	1.141
INC3R	Very limited ability to save	2.45	1.076
INC4R	Need to take additional work to make a living	2.34	1.193
INC5	Income has improved after reform	2.76	1.217
FAIR1	Salary reflects workload	2.48	1.108
FAIR2	Salary is comparable to other professions	2.43	1.106
FAIR3	Allowances reflect the specificity of the profession	2.70	1.165
FAIR4	Seniority-based increments reflect contributions	3.35	1.228
FAIR5	Salary policy is fair	2.59	1.119
COND1R	Remedial teaching is unpaid	1.66	0.730
COND2R	Overtime work is not compensated	1.54	0.712
COND3R	Increasing pressure from parents	1.97	0.877
COND4R	Too many non-teaching tasks	1.76	0.940
COND5R	Curriculum reform increases workload	1.64	0.893
SAT1	Salary policy is reasonable	3.10	1.181
SAT2	Reform attracts young teachers	2.69	0.976
SAT3	Pension policy provides security	3.21	1.289
SAT4R	Salary policy is unlikely to improve	2.31	1.085
SAT5	Feel valued by the state	2.47	1.344
ATT1	Intend to remain in teaching until retirement	3.91	0.832
ATT2R	Would leave for a higher-paying job	3.20	1.255
ATT3	Encourage students to become teachers	3.64	0.949
ATT4	Proud to be a teacher	3.84	0.851
ATT5	Maintain motivation in teaching	3.48	1.075

Source: Survey data, 2026. Reverse-coded items (INC3R, INC4R, COND1R–COND5R, SAT4R, ATT2R) are presented with their original coding before reversal. Higher scores on reverse-coded items indicate stronger disagreement with the statement.

### 4.2 Exploratory factor analysis (EFA)

Exploratory factor analysis extracted five factors from 25 observed variables, explaining a cumulative 62.148% of the total variance. The KMO value of 0.854 and the significant Bartlett's test of sphericity (*p* < 0.05) indicate that the data are suitable for factor analysis. All factor loadings exceed the threshold of 0.4. The five-factor structure is consistent with theoretical expectations, representing: (1) professional attitudes, (2) salary policy satisfaction, (3) working conditions and job pressure, (4) parental pressure and personal income, and (5) perceived pay fairness. The rotated factor space diagram is shown in Figure 2.

**Figure 2:**
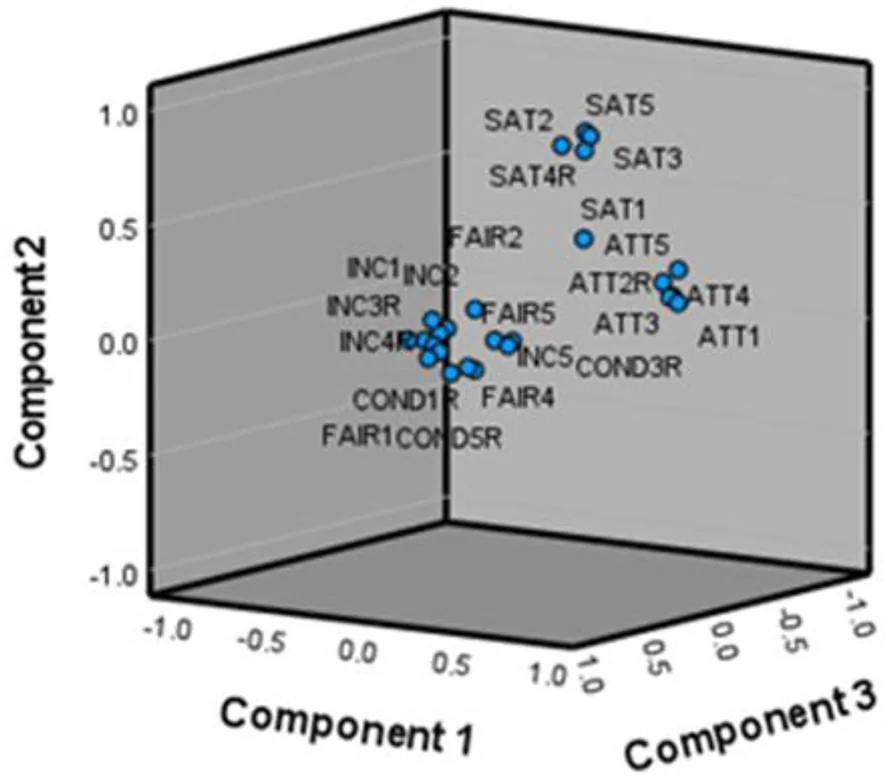
Rotated factor space diagram.

The Component Plot in Rotated Space visually represents the relationships between 25 observed variables and 5 factors after Varimax rotation. The results show that the variables are distributed into five distinct clusters, corresponding to the five factors extracted from the EFA. Specifically, variables belonging to the Professional Attitude (ATT) scale form a tight cluster; variables of the Salary Satisfaction (SAT) scale form a second cluster; Working Conditions (COND) variables form a third large cluster; along with two smaller clusters representing Actual Income (INC) and Comparative Fairness (FAIR). The SAT1 variable is located at the boundary between two factors, explaining its cross-loading in the subsequent CFA. Overall, the diagram confirms that the five-factor structure is appropriate. The EFA results are summarized in Table 4.

**Table 4:** Results of exploratory factor analysis (EFA).

Indicator	Result	Evaluation
KMO	0.854	Excellent
Bartlett's test of sphericity	Sig. = 0.000	Acceptable
Number of factors extracted	5	Consistent with theoretical expectations
Cumulative variance explained	62.148%	Acceptable (>50%)
Factor loadings	0.460 – 0.925	All > 0.4, acceptable
Cross-loadings	Some variables show cross-loadings, but differences are acceptable	Acceptable

### 4.3 Measurement model (CFA)

Confirmatory factor analysis (CFA) was conducted to validate the measurement structure of the research constructs identified through exploratory factor analysis (EFA). The measurement model consisted of five latent factors and 25 observed variables, and was estimated using the maximum likelihood (ML) method with a sample size of *N* = 357. The CFA results allow for the assessment of composite reliability (CR), average variance extracted (AVE), as well as convergent and discriminant validity of the scales. Standardized factor loadings, composite reliability (CR), and average variance extracted (AVE) are presented in Table 3. The CFA measurement model is presented in Figure 3.

**Figure 3:**
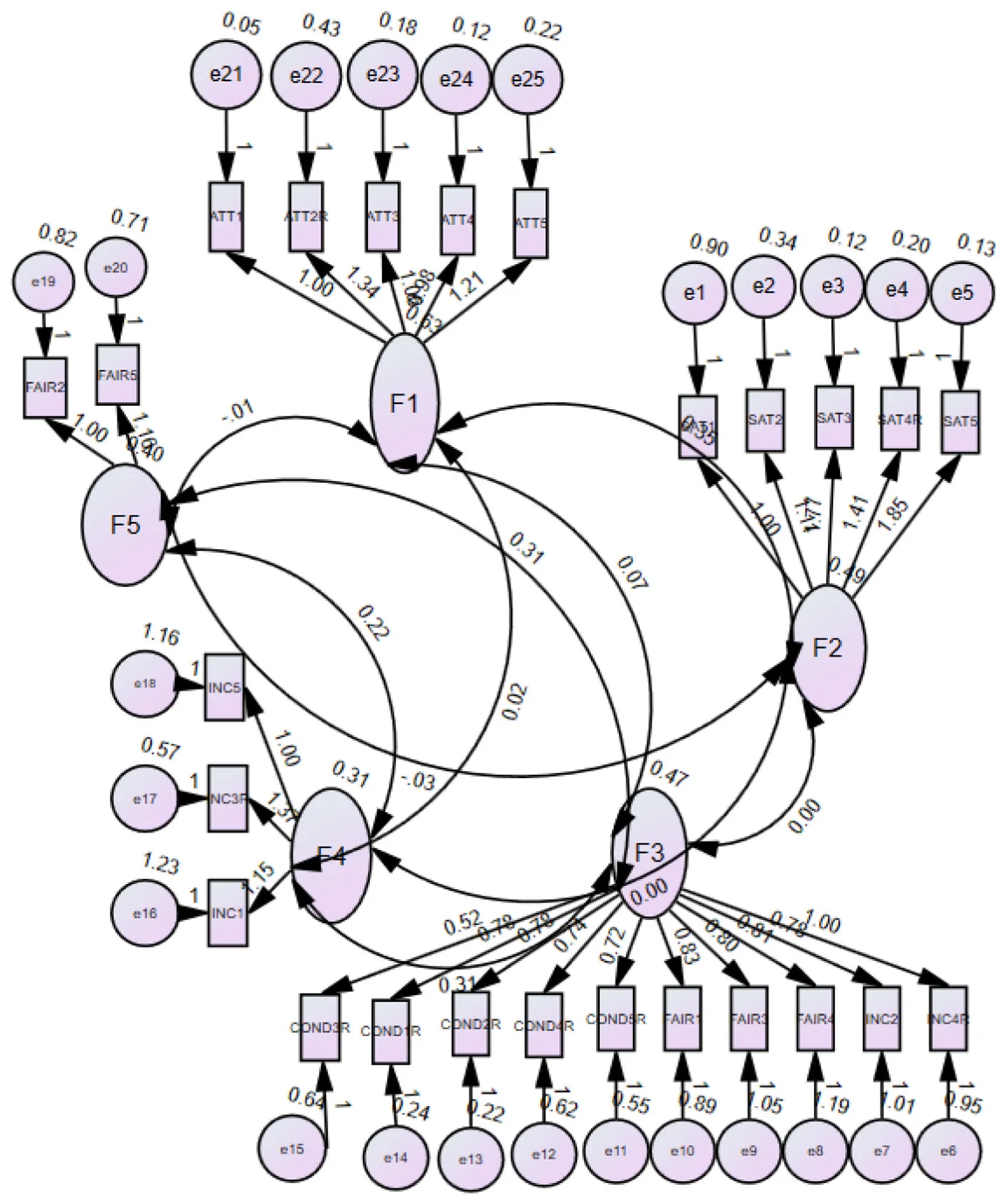
CFA.

Confirmatory Factor Analysis (CFA) was conducted to validate the five-factor measurement model.

#### 4.3.1 Model fit

CMIN/df = 3.782, CFI = 0.873, TLI = 0.856, RMSEA = 0.088 (90% CI: 0.082–0.094), SRMR = 0.078. The low loading of COND3 (0.413) and the low alpha for FAIR (0.630) are acknowledged as limitations. While CFI and TLI were below the ideal threshold of 0.90, the model was considered acceptable given the complexity of the constructs, the sample size, and the fact that RMSEA and SRMR were within acceptable ranges (Hair et al., 2010).

### 4.4 Structural model (SEM)

After completing the CFA, the next step was to conduct structural equation modeling (SEM). The purpose of this analysis was to examine whether relationships exist among the identified factors and to assess the strength of these relationships. Based on the core constructs identified through EFA, SEM was used to test the hypothesized relationships. The squared multiple correlations (R^2^) are reported in Table 6. The structural model with standardized path coefficients is shown in Figure 4.

**Table 5:** Standardized factor loadings, CR, and AVE from CFA.

Construct	Items	Standardized loadings	CR	AVE
SAT	SAT1	0.593	0.931	0.736
	SAT2	0.800		
	SAT3	0.963		
	SAT4R	0.910		
	SAT5	0.965		
COND	COND1R	0.735	0.815	0.314
	COND2R	0.753		
	COND3R	0.413		
	COND4R	0.541		
	COND5R	0.558		
INC	INC1	0.501	0.580	0.323
	INC2	0.471		
	INC3R	0.712		
	INC4R	0.578		
	INC5	0.460		
FAIR	FAIR1	0.519	0.551	0.381
	FAIR2	0.575		
	FAIR3	0.472		
	FAIR4	0.456		
	FAIR5	0.657		
ATT	ATT1	0.959	0.958	0.819
	ATT2R	0.853		
	ATT3	0.894		
	ATT4	0.915		
	ATT5	0.899		

CR, Composite Reliability; AVE, Average Variance Extracted. All loadings are standardized and significant at ^*^p < 0.001.

**Table 6:** Squared multiple correlations (*R*^2^).

Dependent variable	*R* ^2^	Interpretation
SAT (Salary Satisfaction)	0.009	Very low explanatory power
ATT (Professional Attitudes)	0.437	Moderate explanatory power

The model explained 43.7% of the variance in professional attitudes (ATT), indicating that the included variables have good predictive power for teachers' professional attitudes.

**Figure 4:**
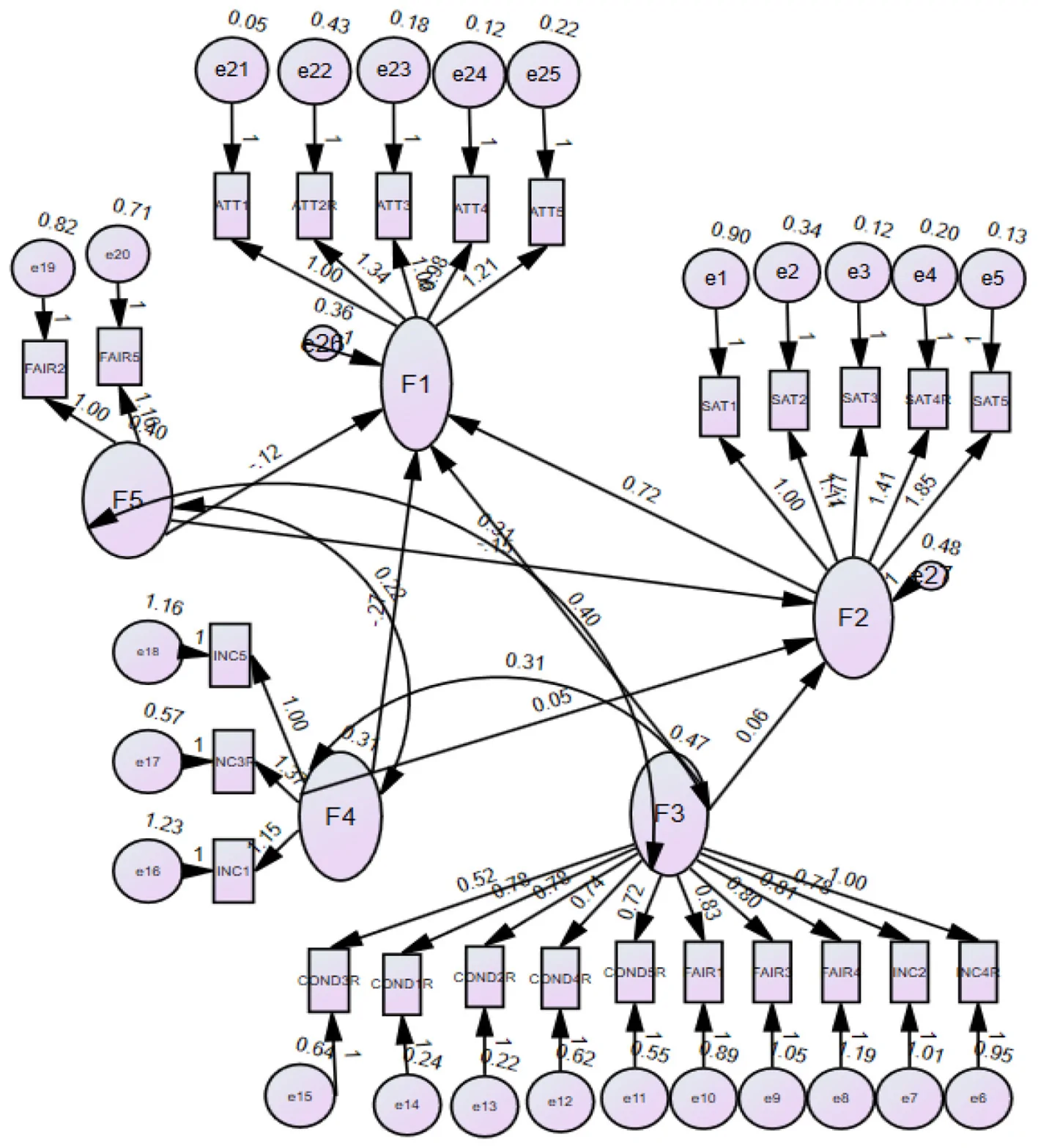
SEM.

The SEM results provide several interesting findings. First, satisfaction with salary policy (SAT) has a strong positive association with professional attitudes (ATT) (β = 0.631, *p* < 0.001). This suggests that teachers who are more satisfied with salary policy tend to show more pride and commitment to their profession. Second, working conditions and occupational pressure (COND) also positively relate to ATT (β = 0.346, *p* = 0.022). Because COND was reverse-coded so that higher scores indicate more positive working conditions (i.e., less unpaid work, less workload, less parental pressure, and less reform-related pressure), this finding indicates that teachers who report more positive working conditions tend to show more positive professional attitudes. This association is consistent with international research showing that better working conditions are linked to higher job satisfaction and commitment (Kyriacou, 2001; Maslach and Leiter, 2016; Hakanen et al., 2006; Toropova et al., 2021). Third, neither actual income (INC) nor fairness in salary policy (FAIR) was significantly associated with SAT or ATT. Specifically, the paths SAT ← INC (β = 0.038, *p* = 0.818), SAT ← FAIR (β = −0.139, *p* = 0.301), ATT ← INC (β = −0.187, *p* = 0.180), and ATT ← FAIR (β = −0.099, *p* = 0.354) were all non-significant. Therefore, H3 and H4 were not supported. The non-hypothesized paths (ATT ← INC, ATT ← FAIR, SAT ← COND) were also non-significant (all *p* > 0.05).

In summary, satisfaction with salary policy and working conditions are the two key correlates of professional attitudes in this sample, while actual income and fairness perceptions appear less influential.

### 4.5 Summary of findings

The SEM analysis revealed three main findings:

(1) Satisfaction with salary policy (SAT) is the strongest predictor of professional attitudes (ATT) (β = 0.631, *p* < 0.001). This finding is consistent with Herzberg's two-factor theory, which posits that salary is a hygiene factor—when inadequate, it causes dissatisfaction, but when adequate, it enables motivation to emerge. In the Vietnamese context, this suggests that improving teachers' satisfaction with salary policy may be a prerequisite for strengthening their professional attitudes, including pride in the profession, intention to stay, and motivation to teach.

(2) Working conditions and occupational pressure (COND) positively relate to professional attitudes (ATT) (β = 0.346, *p* = 0.022). This “paradoxical” finding may reflect the unique cultural context of Vietnam, where teachers take pride in overcoming hardship and view work pressure as part of their professional identity (the cultural value of tôn sư trọng đạo). However, this association needs to be replicated in future research before firm conclusions can be drawn, especially given the cross-sectional design and the coding of COND items.

(3) Actual income (INC) and fairness in salary policy (FAIR) do not significantly relate to SAT or ATT. Specifically, H3 (INC → SAT) and H4 (FAIR → SAT) were not supported (*p* > 0.05). This suggests that teachers' satisfaction with salary policy is shaped less by their actual income level or fairness comparisons with other professions, and more by their perceptions of the policy itself (e.g., pension security, trust in future reforms). However, given the low reliability of the FAIR scale (α = 0.630) and the low AVE values for INC and FAIR, these non-significant findings should be interpreted with caution.

## 5 Discussion

### 5.1 The central role of salary satisfaction

The finding that salary satisfaction (SAT) has the strongest association with professional attitudes (ATT) (β = 0.631) is consistent with previous research. Hanushek (2011) argued that higher teacher salaries attract more qualified individuals and reduce turnover. Ingersoll and Smith (2003) found that low salary is a primary reason teachers leave the profession. In the Vietnamese context, Lê et al. (2025c) found that teacher salary satisfaction remained low despite policy improvements. Our study extends this finding by quantifying the association between salary satisfaction and professional attitudes using SEM. The strong coefficient (β = 0.631) indicates that improving salary satisfaction could be associated with significantly enhanced professional attitudes.

### 5.2 The “paradoxical” effect of working conditions

The finding that working conditions (COND) positively relate to professional attitudes (β = 0.346) appears paradoxical at first glance. International research typically finds that high workload and work pressure are associated with burnout and reduced job satisfaction (Kyriacou, 2001; Maslach and Leiter, 2016; Hakanen et al., 2006). However, this finding may be explained by the unique cultural context of Vietnam. The concept of “tôn su' trọng đạo” (respect for teachers) is deeply embedded in Vietnamese culture. Teachers are traditionally viewed as “ngu'i lái đò” (ferryman) who sacrifice their own comfort to transport students to a better future. This cultural narrative frames hardship and sacrifice as integral to the teaching profession, rather than as external burdens. However, this explanation is hypothetical and was not directly tested in this cross-sectional survey. The result of the working condition is an association that needs to be replicated, not evidence that work pressure leads to pride or work pressure helps ward off burnout. Future research should examine this relationship longitudinally and qualitatively.

### 5.3 The role of actual income and fairness in salary policy

The finding that actual income (INC) and fairness in salary policy (FAIR) do not significantly relate to satisfaction with salary policy (SAT) or professional attitudes (ATT) is notable. This may be explained by several factors.

First, regarding actual income (INC), Vietnamese teachers may evaluate their salary policy not solely based on their current income level, but also on other aspects of the policy such as pension security, stability, and trust in future reforms. In other words, teachers may distinguish between their immediate income situation and their overall evaluation of the salary policy. This distinction is consistent with the low correlation observed between INC and SAT in our data, and it suggests that improving actual income alone may not automatically translate into higher satisfaction with salary policy.

Second, regarding fairness in salary policy (FAIR), Vietnamese teachers may have accepted the income gap with other professions as a reality of the profession. Despite this awareness, teachers do not attribute this gap to salary policy unfairness, perhaps because they value the stability and pension benefits of public employment. Additionally, there may be conceptual overlap between SAT and FAIR, as both involve evaluations of pay. This overlap is reflected in the discriminant validity concerns reported in Section 3.3, where the square root of AVE for FAIR (0.617) was lower than the FAIR–COND correlation (0.718). Future research should examine these constructs more carefully to ensure discriminant validity.

Finally, it is important to note that the FAIR scale showed relatively low reliability (α = 0.630) and low AVE (0.381), which may have attenuated its relationship with SAT and ATT. Similarly, the INC scale showed low AVE (0.323). These measurement limitations should be taken into account when interpreting the non-significant findings, and they highlight the need for further measurement refinement in future research.

### 5.4 Comparison with previous studies

Our findings are consistent with several international studies. Skaalvik and Skaalvik (2011) found that salary satisfaction was a significant predictor of both job satisfaction and turnover intentions among Norwegian teachers. Toropova et al. (2021) found that working conditions—including workload and administrative support—were important predictors of teacher job satisfaction. However, our finding that working conditions positively relate to professional attitudes diverges from most Western studies. This suggests that cultural context may play a role in shaping how teachers perceive and respond to work pressure. In the Vietnamese context, our findings align with Bui and Dinh (2025), who found that intrinsic motivation (love for children) was the strongest predictor of students' choice of pedagogical majors. They also align with Lê et al. (2025c), who found that teacher salary satisfaction remained low due to the gap between basic salary and living costs.

### 5.5 Theoretical contributions

This study makes several theoretical contributions. First, it integrates public policy theory (Chen, 2003; Chen and Garbe, 2011; Chen et al., 2024) with motivation theory (Herzberg, 1959; Vroom, 1964; Adams, 1965) to provide a comprehensive framework for understanding teacher salary satisfaction and professional attitudes. Second, it provides empirical evidence from Vietnam, a country undergoing major salary reform, extending the applicability of these theories to the Southeast Asian context. Third, it identifies the “paradoxical” positive association of working conditions with professional attitudes, highlighting the potential role of cultural factors in shaping teacher motivation. However, this finding needs to be replicated before firm conclusions can be drawn.

### 5.6 Common method bias and control variables

Given that the study used self-reported data from a single source, common method bias may be a concern. Harman's single-factor test was conducted, and the first factor explained 32.4% of the variance, which is below the 50% threshold, suggesting that common method bias is not a major issue. Additionally, we controlled for age, gender, tenure, region, and school location (urban/rural) in the SEM analysis. None of these control variables significantly altered the pattern of relationships reported in Table 7.

**Table 7:** Standardized regression weights from SEM.

Path	β	SE.	CR.	*p*	Hypothesis	Result
ATT ← SAT	0.631	0.073	9.830	^***^	H1	Supported
ATT ← COND	0.346	0.175	2.287	0.022	H2	Supported
SAT ← INC	0.038	0.208	0.230	0.818	H3	Not supported
SAT ← FAIR	−0.139	0.147	−1.035	0.301	H4	Not supported
ATT ← INC	−0.187	0.198	−1.340	0.180	Non-hypothesized	Not significant
ATT ← FAIR	−0.099	0.135	−0.926	0.354	Non-hypothesized	Not significant
SAT ← COND	0.062	0.183	0.345	0.730	Non-hypothesized	Not significant

^***^p < 0.001. H1–H4 correspond exactly to the hypotheses stated in Section 2.5. The three non-hypothesized paths (ATT ← INC, ATT ← FAIR, SAT ← COND) were estimated to check for potential direct effects but are not part of the formal hypothesis testing. INC = Actual Income (F4); FAIR = Fairness in Salary Policy (F5); COND = Working Conditions and Occupational Pressure (F3); SAT = Satisfaction with Salary Policy (F2); ATT = Professional Attitudes (F1).

## 6 Conclusion

### 6.1 Summary of findings

This study examined how satisfaction with salary policy and working conditions relate to the professional attitudes of primary school teachers in Vietnam during the 2024–2026 salary reform. Using SEM analysis of survey data from 357 teachers, the study found:

(1) Satisfaction with salary policy (SAT) has the strongest positive association with professional attitudes (ATT) (β = 0.631, *p* < 0.001). Teachers who are more satisfied with salary policies are significantly more likely to have positive professional attitudes, including pride in the profession, intention to stay, and motivation to teach.

(2) Working conditions and occupational pressure (COND) also positively relate to professional attitudes (ATT) (β = 0.346, *p* = 0.022). This “paradoxical” finding may reflect the unique cultural context of Vietnam, but it needs to be replicated in future research.

(3) Actual income (INC) and fairness in salary policy (FAIR) do not significantly relate to satisfaction with salary policy (SAT) or professional attitudes (ATT). Teachers appear to evaluate salary policies independently of their actual income level or comparisons with other professions, focusing instead on their own needs and expectations regarding the policy itself (e.g., pension security, trust in future reforms).

### 6.2 Policy implications

Based on these findings, several policy implications emerge. These suggestions are offered cautiously, given that the study is cross-sectional and does not directly test the impact of specific policy changes.

First, increasing the basic salary may be considered. The root of the salary problem is the low basic salary. Currently, a newly graduated teacher earns only 5–6 million VND/month, which is insufficient to cover living costs in urban areas. The government could consider raising the starting salary to at least 10–12 million VND/month, allowing teachers to live decently without relying on tutoring income.

Second, reconsidering the proposed abolition of seniority allowances may be warranted. Seniority allowances (5% after 5 years, increasing 1% annually up to 30%) are a crucial factor in retaining experienced teachers. Abolishing this allowance without a replacement mechanism could reduce the income of senior teachers and undermine their motivation. If reform is necessary, a gradual transition period of at least 5–7 years should be provided, with protections for teachers who have already accumulated significant seniority.

Third, leveraging the strength of the pension system may be beneficial. The pension system is highly valued by teachers, especially those with longer tenure. For young teachers, the promise of a stable pension helps them endure current low salaries. The government should maintain and strengthen the pension system, ensuring that it remains a reliable source of retirement income.

Fourth, improving working conditions in disadvantaged areas may be important. Teachers in mountainous and remote areas face unique challenges, including separation from family, high living costs, and limited professional development opportunities. The government could provide housing support, travel subsidies, and priority admission for teachers' children to quality schools.

Fifth, addressing the tutoring ban's impact on teacher income may be considered. Circular No. 29/2024/TT-BGDÐT has cut off a major source of supplementary income for primary school teachers. The government could consider allowing schools to organize afternoon skill-based clubs (music, art, sports, English) where teachers can earn legitimate income while providing valuable services to students.

### 6.3 Limitations and future research

This study has some limitations. First, the sample, while diverse, may not be fully representative of all primary school teachers in Vietnam. Future research should expand the sample to include more teachers from remote areas and private schools. Second, the cross-sectional design limits causal inference. While SEM allows for testing of directional relationships, longitudinal data would provide stronger evidence for causal effects. Future research should track the same teachers over time to examine how salary satisfaction and professional attitudes change as the reform is implemented. Third, the study focused on teacher-reported outcomes. Future research should examine objective outcomes, such as student achievement, to assess the actual impact of salary reform on educational quality. Fourth, the study did not include principals or policymakers as respondents. Future research should incorporate multiple perspectives to provide a more comprehensive understanding of the policy implementation process. Fifth, the low reliability of the FAIR scale and the low loading of COND3 suggest that these constructs may need further refinement in future research.

### 6.4 Conclusion

This study provides empirical evidence that salary satisfaction is the strongest predictor of professional attitudes among primary school teachers in Vietnam. The paradoxical positive association of working conditions highlights the unique cultural context in which Vietnamese teachers take pride in overcoming hardship, but this finding needs to be replicated. The findings have important implications for salary reform policy, emphasizing the need to increase basic salaries, preserve seniority allowances, leverage the pension system, improve working conditions in disadvantaged areas, and address the impact of the tutoring ban on teacher income. For salary reform to succeed, policies must not only be well-designed but also effectively implemented. Teachers need to see tangible improvements in their monthly paychecks before they will fully trust and commit to the reform.

## Data Availability

The datasets generated and analyzed during the current study are available from the corresponding author on reasonable request.
